# Use of Infrared Thermometry to Observe Temperature Variation Associated with the Healing Process in Wounds and Ulcers: TIHUAP Cohort Study Protocol

**DOI:** 10.3390/healthcare11121750

**Published:** 2023-06-14

**Authors:** Mercè Iruela Sánchez, Rosa García-Sierra, Rafael Medrano-Jiménez, Diana Bonachela-Mompart, Natalia Maella-Rius, Esther Soria-Martín, Mar Isnard-Blanchar, Pere Torán-Monserrat

**Affiliations:** 1 Direcció Atenció Primària Metropolitana Nord, Institut Català de la Salut, 08204 Sabadell, Spain; miruela.ics@gencat.cat (M.I.S.); dbonachela.mn.ics@gencat.cat (D.B.-M.); nmaella.mn.ics@gencat.cat (N.M.-R.); esoria.mn.ics@gencat.cat (E.S.-M.); misnard.bnm.ics@gencat.cat (M.I.-B.); 2 Multidisciplinary Research Group in Health and Society (GREMSAS) (2021-SGR-0148), 08007 Barcelona, Spain; ptoran.bnm.ics@gencat.cat; 3 Grup D’experts en Ferides, Institut Català de la Salut GEICS, 08007 Barcelona, Spain; medrano.vascular@gmail.com; 4 Research Institut, Universitari d’Investigació en Atenció Primària Jordi Gol (IDIAP JGol), 08303 Mataró, Spain; 5 Nursing Department, Faculty of Medicine, Campus Bellaterra, Universitat Autònoma de Barcelona, 08193 Barcelona, Spain; 6 Primary Care Group, Germans Trias i Pujol Research Institute (IGTP), 08916 Badalona, Spain; 7 Department of Medicine, Faculty of Medicine, Universitat de Girona, 17004 Girona, Spain

**Keywords:** wound healing, thermography, monitoring, thermal index, chronic wound

## Abstract

We are interested in observing how temperature differences between the wound bed and perilesional skin are related to the healing process in primary care patients with wounds. Multisite prospective cohort study with one-year follow-up in the Metropolitan North area of Barcelona. Recruitment of patients over 18 years with an open wound will take place from January 2023 to September 2023. Temperature checks will be conducted on a weekly basis at control visits and wound care. The following variables will be measured: Percentage reduction of wound area over time, thermal index, the Kundin Wound Gauge, and the Resvech 2.0 Scale. The temperature will be measured weekly using a handheld thermometer and mesh grid to frame the temperature points. The healing trajectory will also be monitored on a monthly basis via photographic imaging, the Resvech Scale, calculation of wound size, percentage reduction of wound area over time, and thermal index for one year of follow-up or until the wound is cured. This study may represent a turning point for its introduction into primary care. Early diagnosis of wound complications would facilitate treatment decision-making for healthcare professionals, thus improving the management of resources related to chronic wounds.

## 1. Introduction

Ulcers are defined as “a lesion on the surface of the skin or a mucous surface, produced by the sloughing of inflammatory necrotic tissue”. The physiological healing process consists of different phases: hemostasis, inflammation, proliferation, migration, and, lastly, remodeling. Wounds are classified by the duration of the healing process, with short-term wounds being those that heal in under six weeks and chronic wounds taking longer than six weeks to heal [1,2].

In primary care (PC), the most common types of chronic ulcers are venous (VU) resulting from chronic venous insufficiency, dependence-related lesions (DRL), including those caused by pressure (PU), shear and humidity [3], and, lastly, diabetic foot (DFU) stemming from diabetes mellitus [2]. The prevalence of chronic ulcers has increased as the population has aged, and comorbidities have become more abundant. Internationally, it is estimated that the prevalence of chronic wounds of various etiologies is 2.21 per 1000 [4]. The latest study published by the National Group for the Study and Assessment of Pressure Ulcers and Chronic Wounds (GNEAUPP) [5] established that PUs in the hospital setting remained stable between 7 and 8% while they increased from 7.9 to 9.1% in home care and from 12.6 to 14.2% in social health centers, as compared to the previous study [6].

Treating chronic wounds has a high economic and human cost, although it varies depending on the type of ulcer and care setting. Lesions that take longer than six months to heal cost twice as much as those with a shorter healing time [7]. Díaz Herrera (2013) analyzed the annual cost of materials for treating chronic ulcers of various etiologies in 17 PC and social health centers. The study estimated that the total cost for primary care centers ranged from € 218,740.95 to € 722,350.05 [8]. According to a study by Soldevilla in 2011, the cost of ulcers in Spain accounts for 5.2% of total health spending, which falls very closely in line with other countries, 5.5% [6,9].

Various recommendations have been made regarding the proper prevention and management of chronic ulcers depending on the risk of lesions and the functional status of the patient. However, there is no gold standard for chronic ulcer prevention and management. Several guidelines have been published on the use of risk assessment and healing scales for these ulcers and the functional status of the patient [10,11]. In 2011, Restrepo-Medrano published a new assessment scale called Resvech 2.0. This scale can be applied to all types of chronic ulcers and targets six wound assessment dimensions: size, depth, tissue type, state of edges and skin, exudate, and signs of infection (inflammation, pain, heat, erythema). Total scores on the Resvech 2.0 scale range from 0 to 35 [12].

The Cochrane Institute published a systematic review on the evaluation of 19 interventions for the prevention of foot ulcers in people with diabetes. Of these, the ones that obtained the greatest relevance due to their beneficial effects were silicone orthoses, infrared thermometry, shoe size, and shock-absorbing insoles [13].

In other studies, different non-contact and non-invasive interventions are proposed, such as fluorescence imaging or harmonic ultrasound, considering laser point imaging as the most powerful intervention [14].

Temperature is a physiological thermoregulatory mechanism against infection and ischemia [15,16,17,18] and increases in the area of local inflammation. It may be another variable to consider in wound assessment since the difference between intralesional and perilesional temperature could be a predictor of the onset of infection in the wound [8].

Thermometry has been used to monitor diabetic foot ulcers in various studies. A systematic review published in 2017 concluded that infrared thermometry is a non-contact method that can assist in the early detection of diabetic foot complications and the administration of early treatment [19].

The use of infrared thermography has increased in recent years, especially in hospitals, due to the advantages it poses over other methods [19]. Nevertheless, thermographic cameras are expensive and require specific training to interpret results.

There are various methods to describe the surface of an ulcer: digital planimetry, photography, and acetate. More simple and affordable options include the Kundin Wound Gauge, which calculates the percentage reduction of wound area over time [18] and local temperature. Currently, local temperature can be measured using non-contact handheld infrared thermometers. This technique can detect subtle temperature variations that go undetected by touch [19]. This is a much more affordable alternative to infrared thermography and can also be used by people without professional training, which would allow patients to monitor temperature at home [20].

The difference between intralesional and perilesional temperature as a prognostic value in wound healing was proposed by Bharara through the calculation of the thermal index (TI), also referred to as the wound inflammatory index [18]. It has been demonstrated that the thermal index value of a healing wound will be closer to 0 than a non-healing wound (Yee 2018) [21]. According to Maliyak 2020, increased local wound temperature combined with two or more infection criteria is an indicator of deep infection [22].

Infrared thermometry is a non-invasive exploratory technique that measures the amount of heat emitted by the body using instruments that capture this radiation and express it numerically [19]. Infrared thermometers yield reliable temperature readings, which, along with other signs, make it possible to detect deep infections in tissue lesions [23]. Most studies that have applied infrared thermometry to the monitoring, prevention, and prognosis of chronic ulcer healing have been carried out in hospitals [24,25]. To the extent of our knowledge, there is one initiative that used thermometry to monitor diabetic foot in primary care [26]. The most noteworthy strength of infrared thermometry is that it can be applied in any care setting and by non-professional personnel with minimal training, which makes it an ideal technique for use in any care setting, including home care.

It must be demonstrated that temperature is a factor that assists in understanding the trajectory of short-term wounds and the physiological and pathological processes involved in chronic ulcers [17,27] so that the possibility of incorporating this tool into care plans may be explored [28,29].

### Aims

The aim of the study is to observe how temperature differences between the wound bed and the perilesional skin are related to the wound healing process in primary care patients.

The specific objectives are:

- To determine if there is consistency between the size of the lesion as measured with the equation for the percentage reduction of wound area over time and the size category of the Resvech 2.0 scale.
- To identify the concordance between temperature differences between the perilesional skin and the wound bed and the risk of infection category on the Resvech 2.0 scale.

## 2. Materials and Methods

### 2.1. Design

The proposal is for a multisite prospective cohort study with a one-year follow-up of patients with wounds treated by primary care nurses. Participants’ temperatures will be taken using thermometers at the weekly control visit and wound care.

### 2.2. Study Settings

The study will be conducted in the Metropolitan North area of Barcelona, which has a population of 1,342,947, distributed across 64 primary care centers.

### 2.3. Participants

Participants will be recruited at primary care centers in the abovementioned region from January 2023 to September 2023 and will be followed for one year, with the study concluding in September 2024.

Participants will be chosen consecutively, provided that they meet the following inclusion criteria:

- 18 years of age or older at the time of joining the study.
- Presenting one or more wounds with a surface area greater than 1 cm^2^ and less than 34 cm^2^ anywhere on the body surface.
- Willing to sign the written informed consent.

Patients will be excluded based on the following criteria:

- Presenting wounds with a tumoral, infectious, arterial etiology, burns, extensive cellulitis, or exudative edemas.
- Having a disease with a life expectancy of less than 18 months.

### 2.4. Variables and Instruments

The following response variables will be measured:

- Prediction of the healing process based on lesion size changes.
- Healing index
- Healing process, based on temperature differences.

Table 1 presents all the variables that will be measured in the study with their corresponding measurement instruments.

**Table 1:** Variables and measurement instruments.

Variables	Instruments
Prediction of the healing process based on changes in lesion size.	Percentage reduction of wound area over time. Calculated as VA = A0 − A1/t. Where Av is the variation of the lesion area, A0 is the reference control area (1st calculation of Kundin M0), A1 is the monthly control area (M1, M2…), and t is the time variable between A0 and A1 expressed in days [18].
Healing index	Resvech 2.0 scale [12]
Healing process based on temperature differences.	Thermal index (TI) [18] TI = (([intralesional_median] − [ perilesional_median]) × [isotherm_area])/[monthly_kundin]
Temperature point coordinate	Grid available in 3 sizes with coordinates (Figure 1).
Temperatures (wound bed, perilesional, body)	Hand-held, 4-in-1 infrared thermometer, IDOIT brand.
Wound size	Kundin formula. Calculated as: length × width × 0.785 (cm) Performed using a metric template, available for download on the institution’s intranet.
Photograph of wound	Canon PowerShot 1300 16-megapixel camera (Canon, Tokyo, Japan). Taken without flash.
Comorbidity	Dyslipidemia, high blood pressure, heart disease, venous insufficiency, nephropathy, thyroid disorders, peripheral artery disease, immunodeficiency, diabetes mellitus.
Sociodemographic and clinical data	Sex, age, weight, body mass index, limited mobility, physical activity (sufficiently or insufficiently active)
Dependence level	Barthel Index, which assesses 10 activities with scores between 0 and 100, with 0 being maximum dependence and 100 being maximum independence [30].
Nutritional status	Mini Nutritional Assessment (MNA), a questionnaire with 6 items and scores between 0 and 12, with 0 being normal nutritional status and 12 malnutrition status [31].
Wound-related variables	Type of lesion (open wound, surgical dehiscence, sebaceous cyst debridement, pilonidalcystectomy, pressure ulcer, vascular ulcer, diabetic foot ulcer.) Infection of the wound prior to study Prior antibiotic treatment of the wound Wound healing time expressed in days (from the start of the wound to the day of inclusion in the study). Need for wound debridement.

**Figure 1:**
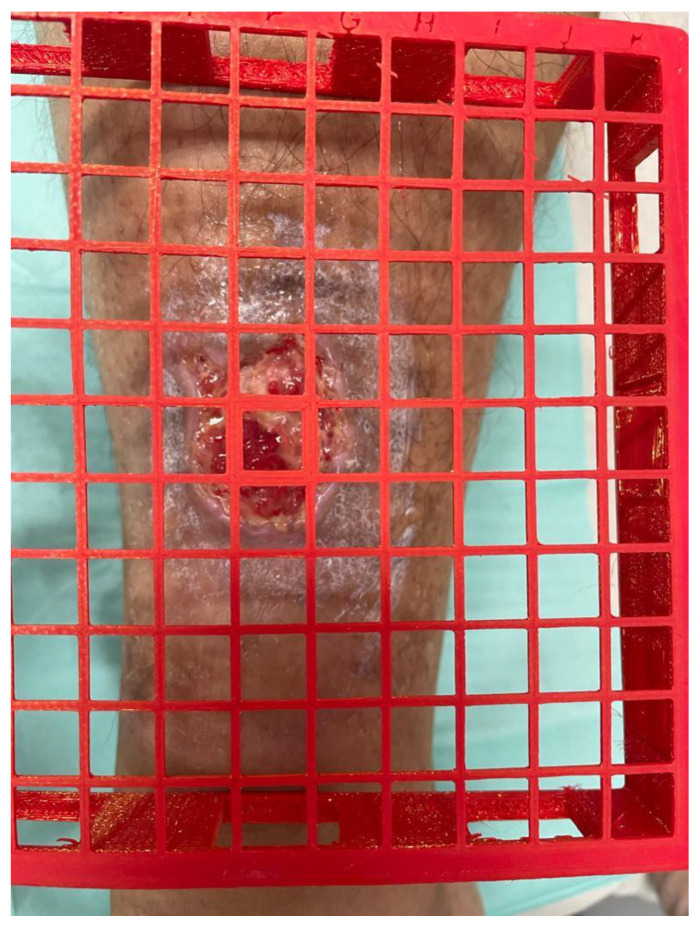
Grid to measure the temperature in the same location.

### 2.5. Recruitment, Consent and Data Collection

The project’s PI held meetings with the directors of the PC centers to present the study and request their collaboration. Later, meetings were held with the primary care nurses who will be in charge of recruiting and explaining the research objectives and inclusion/exclusion criteria. Lastly, a pilot test was conducted on five patients with a follow-up period of four weeks to be able to adapt the methodology and implement the project. All the nurses participating in data collection were provided with detailed written information and the necessary material. The research team will visit the data collection centers periodically to supervise the development of the research.

Purposive sampling will be used, offering patients who attend scheduled nursing visits the option to participate. An effort will be made to obtain a representative sample by including typical groups based on wound type criteria.

### 2.6. Study Procedures

Patients will be recruited at scheduled nursing visits at health centers or homes. Nurses will inform patients about the study and will refer them to the collaborating nurse conducting the study.

At the first visit, the lesion will be identified and classified. The following will be performed: the Resvech 2.0 scale, calculation of the Kundin Wound Gauge using a metric template to determine the size of the lesion (the size of the mesh grid will be chosen based on the size of the lesion), wound imaging via digital photography, and the temperature points on the mesh grid. The following temperature measurements will be taken: control temperature on the forehead and the points planned by the researcher.

Before taking the temperature, the patient will be placed in a supine position on the stretcher for 10 min to avoid errors in the measurement, as well as to acclimatize to the ambient temperature of the room. Special attention will be paid to maintaining a relaxed and calm environment while the nurse prepares the necessary material for the intervention.

Prior to measuring the temperatures, the thermometer will be activated for its self-adjustment in temperature and humidity. Afterward, the temperature measurement will be carried out on the forehead and planned points of the wound, through the mesh grid, at a distance of 2 cm.

Once the dossier, medical history review, and assessment of the secondary variables have been completed, the photograph of the wound will be downloaded and applied to the powerpoint template (ppt) as the initial image. Marking of the planned temperature points ppt template. Identification of the initial image as M0 (initial image).

Follow-up will last for a maximum period of 12 months. This period may be less if the lesion heals sooner (healing is defined as total closure of the epidermis). Weekly visits will be conducted to take the various temperature using a handheld infrared thermometer and the mesh grid. The temperature will be taken at the planned points according to the established order. At these visits, any variations in temperature will be detected, including any increase in the difference between the perilesional and wound bed temperatures. A change in temperature may predict the onset of complications: infection or wound chronicity.

At the monthly visits, in addition to the corresponding weekly intervention (temperature measurements), the healing trajectory will be monitored. This will include photographing the wound, completing the Resvech 2.0 scale, calculating the size of the lesion (the Kundin Wound Gauge), and the percentage reduction of wound area over time. The photograph of the wound will be downloaded and applied to the powerpoint template, identified as M1 (month 1), M2 (month 2) … up to M12 (month 12), or earlier if the wound closes entirely.

In the final evaluation of the process, we will have the scores from the interventions: comparison of the initial temperature scores (ITS) (M0) of the evaluation with the final temperature scores (FTS) (M12 or end of the process), the wound area, and the initial and final Resvech V2.0 scale scores. Data will be collected in a REDCap database [32].

Table 2 shows the timeline of data collection to be performed at each visit.

### 2.7. Sample Size Calculation

According to a study conducted in the Directorate of Primary Care of the Metropolitan North (DAPMN) in 2015, the prevalence of wounds is 2.2. Based on a prevalence of 0.22 in patients with a wound, we calculate that a random sample of 140 patients will be sufficient for an estimate with a confidence level of 95%, with *p* = 0.5 population heterogeneity and a sample error of 8%.

### 2.8. Validity and Reliability

The reliability of the temperature measurements may be compromised by the difficulty in ensuring precise measurement points. To overcome this limitation, the research team collaborated with an engineer to create a 3D-printed mesh grid made of polylactic acid (PLA) for individual use by each patient. PLA is a thermoplastic made from renewable resources such as corn starch, tapioca root, and sugar cane, unlike other industry materials made primarily from petroleum. This material allows us to carry out good disinfection; it is harmless to the patient. Additionally, it provides enough flexibility to adapt the mesh grid to different anatomical shapes where the wound is located. This mesh grid will be available in three sizes (11 × 11 cm, 15 × 15 cm, and 17 × 17 cm) so that it can be adjusted to the size of each ulcer. The edges of the grid will be 2 cm thick, thereby raising the mesh from the skin to prevent direct contact and ensure an exact distance to the measurement point. This instrument will make it possible to record and use the temperature point coordinates at each wound healing visit, thus ensuring that the temperature can be taken in exactly the same location. Figure 1 shows a photograph of the grid.

The influence of room temperature is a variable that could confound the results. It is not feasible to set a standard room temperature for all participants due to the scope of the study (various primary care centers and patient homes). To compensate for this limitation, the patient’s forehead temperature will be the control temperature. It will be taken with the same thermometer before taking the local temperatures. This value will be taken into account during the data analysis.

Additionally, and to avoid any other information bias, the wound will be photographed periodically using a 16-megapixel digital camera, storing the photos on a 64 GB memory card. The photographic record will be added to the database to visually monitor the healing trajectory.

### 2.9. Statistical Analysis

Kruskal–Wallis H test will be used to calculate the temperature difference between the various points of the lesion.

The intraclass correlation coefficient (ICC) will be used to assess agreement between continuous outcome variables.

The influence of sociodemographic and clinical factors on the variation of the temperature difference will be analyzed by means of multiple linear regression adjusted for sociodemographic variables.

The analyses will be carried out with bilateral contrast and an alpha risk of 0.05%. The program SPSS 25 (IBM, Armonk, NY, USA) will be used.

## 3. Expected Results

The results of this study will be the existence of a temperature difference between the perilesional skin and the intralesional tissue, which will predict some type of complication. The secondary result will be the correlation of the temperature difference with the Resvech 2.0 Scale, which indicates a prognosis of the wound healing trajectory. Another expected result is a detailed description of the type of wounds treated, their evolution, and prognosis using the Resvesch 2.0 Scale. Further, diagnostic and socioeconomic factors related to wounds will be pointed out. Dependence and socioeconomic status are characteristics that have a great influence on the wound-healing process, as it is shown in the latest TIMERS document [33].

## 4. Discussion

Various studies have considered the application of thermometry in wound monitoring, many at hospitals [17,22,23,29,34] and few in primary care [24,26]. Our study may represent a turning point for the incorporation of thermometry in primary care.

Another strength of our study is that the temperature points are marked with a grid. In other studies, these points were either not marked [17], or they were marked using thermographic cameras [19,21,23,28,29,34] or costly programs requiring specific management and training. These options are not feasible in the current care primary setting.

An important contribution of the application of thermometry to the follow-up of wounds is the early detection of infection. Early detection would allow the early establishment of local treatment, reducing the prescription of antibiotics. All wounds are colonized by microorganisms; Hansson et al., observed that 86% of chronic wounds without signs of infection have at least one bacterium. This issue has been extensively studied by different studies, which conclude with the importance of the presence of microorganisms in non-healing or in delaying it [35].

One of the great problems of our time is resistance to antibiotics, Cosgrove et al. [36] demonstrated that organisms resistant to antibiotics cause between 1.3 and 2 times more infections than sensitive bacteria.

Wound infection and chronicity are major issues that healthcare professionals deal with on a daily basis. A multitude of interventions to prevent, detect and treat these complications has been described internationally [1,2,8,25]

Demonstrating that thermometry is a useful technique in monitoring the trajectory of wounds in primary care would make it possible to incorporate a tool that yields an objective measurement into the wound healing process. This measurement would facilitate early diagnosis of wound infection and prevent ulcers from becoming chronic. Early diagnosis of wound complications would facilitate treatment decision-making for healthcare professionals, thus improving the management of resources related to long-term wounds [19].

The results of the present research facilitate the personalization of treatments, as well as a reduction in costs, and will open these lines of future research based on the results.

## 5. Conclusions

In conclusion, the implementation of this wound healing monitoring system would be an improvement that complies with the recommendations of clinical practice guidelines. Information hitherto unknown in PC would be obtained, thus reinforcing the decision-making of healthcare professionals for better management of material and human resources related to the problem of chronic wounds and ulcers.

Due to the innovative nature of this study, the publication of the results will represent a great contribution to the scientific community and will also serve as a basis for future studies aimed at improving our knowledge of this technique.

## Figures and Tables

**Table 2:** Visit schedule.

Activities	Recruitment	1st Visit	Weekly	Monthly	Final
Information	X	X			
Informed consent		X			
General data		X			
Photograph		X		X	X
Resvesh 2.0 scale		X		X	X
Kundin Formula		X		X	X
Percentage reduction of wound area over time				X	X
Control temperature		X	X	X	X
Wound bed temperature		X	X	X	X
Perilesional temperature		X	X	X	X
Thermal index				X	X

## Data Availability

No data have been created.
